# Modified Pulsatillae decoction inhibits DSS-induced ulcerative colitis in vitro and in vivo via IL-6/STAT3 pathway

**DOI:** 10.1186/s12906-020-02974-9

**Published:** 2020-06-09

**Authors:** Shaohua Huangfu, Renjie Dou, Sixia Zhong, Mengjie Guo, Chunyan Gu, Artur Jurczyszyn, Ye Yang, Bin Jiang

**Affiliations:** 1 Nanjing Hospital of Chinese Medicine Affiliated to Nanjing University of Chinese Medicine, Nanjing, China; 2 School of Medicine & Holistic Integrative Medicine, Nanjing University of Chinese Medicine, Nanjing, China; 3 Department of Hematology, Jagiellonian University Medical College, 30-051 Cracow, Poland; 4 Laboratory for Combination of Acupuncture and Chinese Materia Medica of Chinese Ministry of Education, Nanjing University of Chinese Medicine, Nanjing, China

**Keywords:** Modified Pulsatillae decoction, NCM460, DSS, Ulcerative colitis, IL-6/STAT3 pathway

## Abstract

**Background:**

Ulcerative colitis (UC) is a chronic inflammatory disorder of the colon and rectum, which is positively correlated with the occurrence of IBD-related colorectal cancer (IBD-CRC). Conventional therapies based on drugs such as corticosteroids, mesalamine, and immunosuppression have serious side effects. Pulsatillae decoction (PD) served as a classical prescription for the treatment of colitis in China, has been shown to exert prominent curative effects and good safety. Based on clinical experience and our amelioration, we added an extra herb into this classical prescription, but its therapeutic effect on UC and the underlying mechanism are still unclear.

**Results:**

We first found the curative effect of modified PD on dextran sodium sulfate (DSS)-incubated NCM460 cells. Then C57BL/6 mice were administered DSS to induce UC to evaluate the therapeutic of modified PD. The results showed that modified PD alleviated the inflammatory injury, manifested in body weight, colon length, and disease activity index, with histological analysis of colon injury. Transcriptomic sequencing indicated that modified PD treatment downregulated the IL-6/STAT3 signaling pathway, and reduced the levels of p-NF-κB, IL-1β and NLRP3, which were confirmed by western blot.

**Conclusions:**

Collectively, our results indict that modified PD could efficiently relieve clinical signs and inflammatory mediators of UC, providing evidence of the anti-colitis effect of modified PD, which might provide novel strategies for therapeutic intervention in UC, which may be applied to the prevention of IBD-CRC.

## Background

Inflammatory bowel disease (IBD), including ulcerative colitis (UC) and Crohn’s disease (CD), is a chronic and recurrent inflammatory disorder of unknown etiology [1]. Its duration and severity are positively correlated with the occurrence of IBD-related colorectal cancer (IBD-CRC) [2]. A recent comparative study in China and Canada showed that compared with CD, the proportion of UC in China was significantly higher among patients with IBD-CRC [3]. The global burden of UC is rising, including the associated healthcare and societal costs. According to US data, the national annual direct and indirect costs related to UC are estimated to be $8.1 billion–$14.9 billion with a prevalence of 238/10000 [4, 5]. Although studies of the Chinese population have shown that the incidence of UC has recently increased, with the economic development and improvement of living standards, the incidence is 1.09–1.64 / 100,000 [2, 3]. Hitherto the precise cause of UC is unknown, recent research indicates that the individual’s genetic sensibility, external environment and commensal microflora are all involved and functionally integrated in the pathogenesis of UC [4, 5]. It is reported that patients with UC express high levels of immunocytokines such as TNF-α and IL-6 [6].

The goal of clinical treatment is to achieve disease remission, prevent disease-related complications, and improve the quality of patients’ life. In the past decades, the conventional therapies for UC have been based on the use of corticosteroids, mesalamine and immunosuppressive drugs. Unfortunately, nearly one-third of the patients who are prescribed steroids requires repeated dosing or persists with refractory disease [7]. Currently biological therapies especially tumor necrosis factor (TNF) inhibitors such as infliximab (IFX), adalimumab (ADA), certulizumab pegol and golimumab start to matter. However, anti-TNF therapy has been accompanied with a certain number of side effects including the risk of serious infections and the occurrence of fatal T cell lymphoma on account of rapid decrease of the T-cell population in the gut tissue [8]. Therefore, there is an urgent need to develop safe and effective therapies for treating UC.

Herbal medicine, the most common modality of complementary treatment, exhibits the abilities of bacteriostasis, anti-inflammation, and anticancer, which has already been used for treating some diseases since the third century B.C. in China. It has emerged as the alternative treatment for inflammatory diseases of late years, including UC. Several studies have shown that herbal medicine and its extracts exert anti-UC effects in vitro and in vivo [9–11]. Pulsatilla decoction (PD) was first prescribed by ancient Chinese physicians Zhang Zhongjing in his medical book “Shang Han Lun”, approximately 1800 years ago. Studies have indicated that PD has multiple therapeutic functions including the anti-*C. albicans*, anti-diarrhea and anti-inflammatory activity [12–14]. However, our previously experiment showed that therapeutic effect of PD on UC was unsatisfactory. The Traditional Chinese Medicine pathogenesis of UC is “combination of excess and deficiency”, its therapeutic medication should be a combination of “dispelling pathogenic factors and strengthening vital energy”. The effect of the initial formulation of Pulsatillae decoction is clearing away heat, detoxify and cooling blood. Based on clinical experience and our amelioration, we added an extra herb, Rhizoma Atractylodis Macrocephalae, to strengthen vital energy, but its therapeutic effect on UC and the underlying mechanism are still unknown.

In this study, Dextran sulfate sodium (DSS)-induced colitis mice model, which is characterized by the morphologically and biochemically, was utilized to investigate the therapeutic effect of modified PD, providing evidence of the utilization of modified PD for treating UC [10]. Furthermore, the study on mechanism also elucidated that IL-6/STAT3 signaling pathway was involved in the action of modified PD for alleviating UC.

## Results

### Modified PD alleviated DSS-induced injury in NCM460 cells

**Fig. 1:**
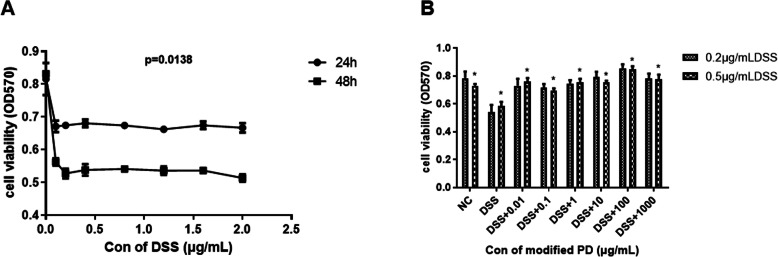
Modified PD ameliorated DSS-induced cell injury on cell viability (A) NCM460 cells were incubated with various concentrations of DSS for 24 and 48 h and their viability were determined by MTT assay. (B) Cell viability of NCM460 treated with different dosages of modified PD and DSS was analyzed by MTT assay. *: p < 0.05; ***: p < 0.001. Data are expressed as mean ± SEM.

To explore the effect of modified PD on DSS-induced UC, we first utilized the NCM460 cell line to evaluate the effect of DSS on cell viability. Cells were treated with a series of concentrations of DSS for 24 h and 48 h, and the cell viability was determined by MTT assay. As shown in Fig. 1A, we found that DSS significantly decreased cell viability and exerted its most deleterious effect at a minimum concentration of 0.2 μg/mL. Meanwhile, to assess the therapeutic effect of modified PD against the cell damage induced by DSS, we performed the exposure of NCM460 cells to modified PD and DSS simultaneously. Results showed that modified PD treatment ameliorated cell viability, and exerted the most effective action at 100 μg/mL (Fig. 1B). These data indicated that modified PD alleviated DSS-induced injury in NCM460 cells.

**Table 1:** Formulation of modified PD

	ingredient	full taxonomic name
1	*Pulsatillae Radix*	*Pulsatilla chinensis (Bge.) Regei*
2	*Fraxini Cortex*	*Fraxinus rhynchophylla Hance*
3	*Phellodendri Chinensis Cortex*	*Phellodendron chinense Schneid.*
4	*Coptidis Rhizoma*	*Coptis chinensis Franch.*
5	*Atractylodis Macrocephalae Rhizoma*	*Atractylodes macrocephala Koidz.*

**Table 2:** Primers used in Quantitative real-time PCR (qRT-PCR)

Target genes	Forward (5′-3′)	Reverse (5′-3′)
TNF-α	TCTACTCCCAGGTCCTCTTCAAG	GGAAGACCCCTCCCAGATAGA
IL-6	GTAGTGAGGAACAAGCCAGAGC	TACATTTGCCGAAGAGCCCT
VEGF	GGAGGGCAGAATCATCACGA	GCTCATCTCTCCTATGTGCTGG
GAPDH	GTCGGAGTCAACGGATT	AAGCTTCCCGTTCTCAG

### Modified PD relieved DSS-induced UC in vivo

**Fig. 2:**
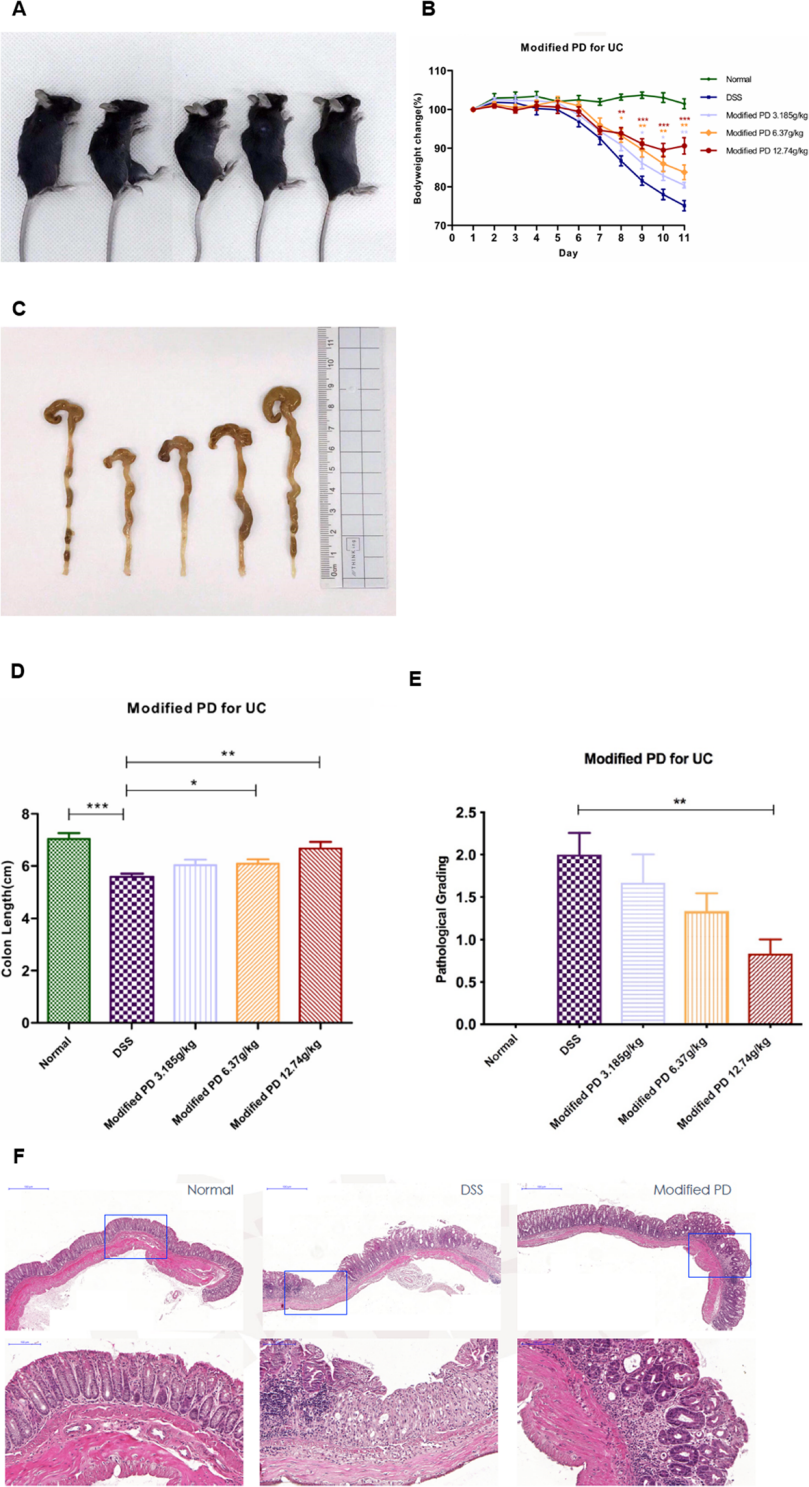
Modified PD administration alleviated symptoms of DSS-induced UC (A) Mice were divided into negative control group, model group (DSS-treated) and three modified PD groups (DSS-treated with modified PD treatment): low dose (3.185 mg/kg), medium dose (6.37 mg/kg) and high dose (12.74 mg/kg). (B) Loss of basal body weight, (C & D) colon length, (E) Pathological score, and (F) HE staining was performed to evaluate the therapeutic effect of modified PD. *: p < 0.05, **: p < 0.01, ***: p < 0.001, Data are expressed as mean ± SEM.

To further determine the protective role of modified PD against DSS-induced UC, we then explored whether modified PD could exert similar effect in vivo. Modified PD was given to an animal model of UC induced by DSS. As presented above, C57BL/6 mice were given 2.5% DSS (w/v) in their drinking water for the induction of acute UC and were simultaneously administered modified PD at a concentration of 3.185, 6.37 and 12.74 g/kg for 10 days (Fig. 2A). Body weight was recorded every day and the animals were sacrificed at 11th day, after which their colons were stored in formalin. As shown in Fig. 2B, the greatest weight loss was observed in the group treated with DSS alone, while the body weight in control group was almost unchanged. Mice given modified PD experienced less weight loss than those given DSS alone, which was closely related to the drug dose. The colon length and pathological grading also demonstrated the above conclusion (Fig. 2C-E). Additionally, H&E staining of the colon sections indicated that modified PD significantly abolished the immunological injury in DSS-treated colon tissues (Fig. 2F). Taken together, these results showed that modified PD ameliorated DSS-induced UC in vivo.

### Transcriptome sequencing (RNA-seq) analysis hinted the potential signaling pathway involved in DSS-induced UC with modified PD treatment

**Fig. 3:**
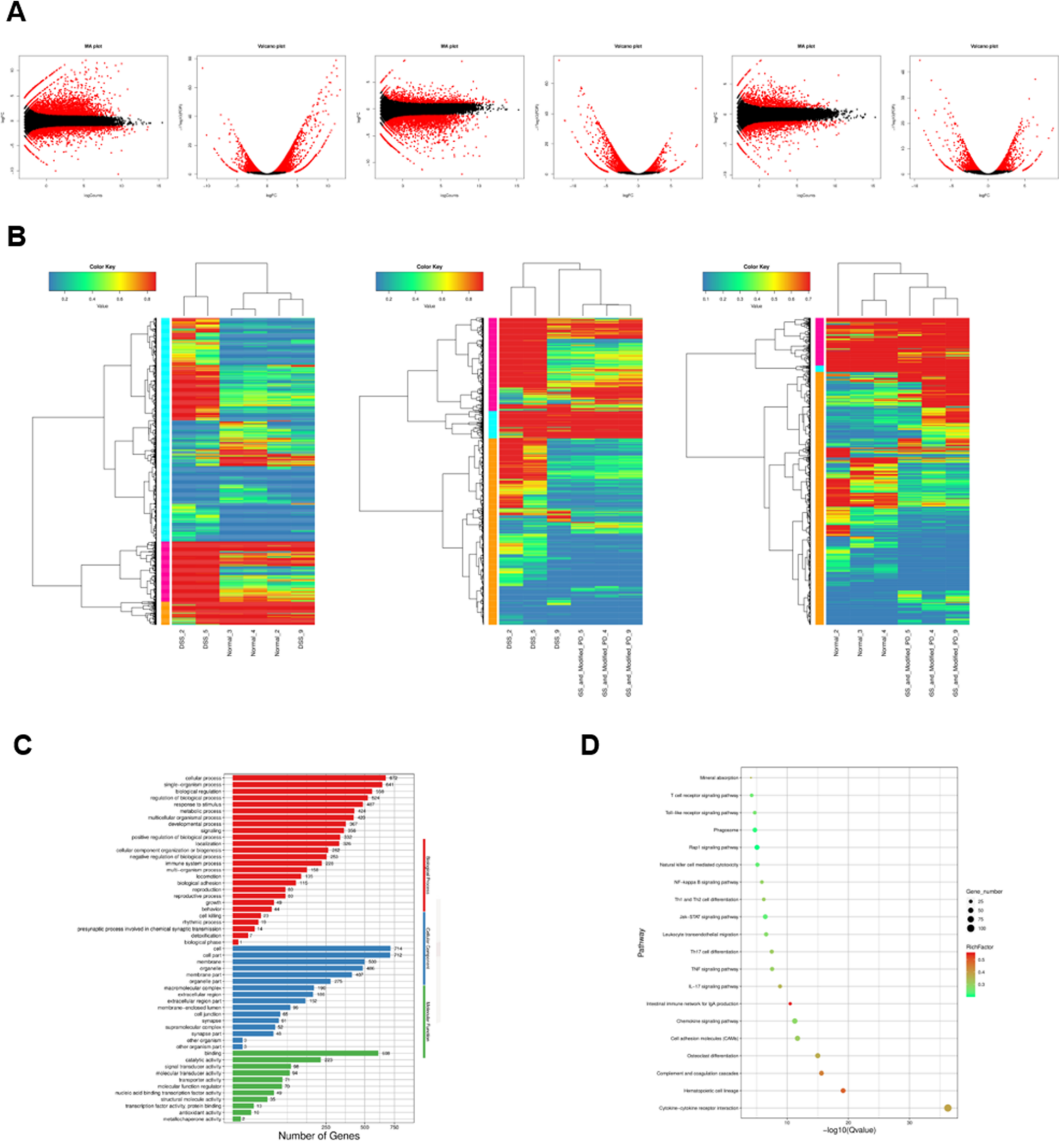
High-throughput transcriptomic sequencing of negative control group, model group and modified PD group (n = 3 in every group) (A) The MA plot and volcano plot were used for showing variance in gene expression with respect to fold change (FC) and significance (p-value). Each dot represents an individual gene: The red point in the plot represented significantly upregulated or downregulated RNA (fold change> 2, P < 0.05) and the black point demonstrated RNA with no statistical differences (fold change< 2, P > 0.05) (Left: negative control group vs. model group; Middle: model group vs. modified PD group; Right: negative control group vs. modified PD group). (B) Microarray heat map demonstrates clustering of colon tissues (Left: negative control group vs. model group; Middle: model group vs. modified PD group; Right: negative control group vs. modified PD group). (C) GO classification of target genes. The 30 most significantly enriched GO terms for colon tissues between model group and modified PD group. (D) KEGG pathway classification of target genes. The Y-axis showed the name of pathway and the X-axis represented the rich factor. The point size indicated the number of differentially expressed genes in one pathway, and the color of the point corresponded to the range of the Q value. The 20 most significant up-regulated pathways (model group vs. modified PD group).

To determine the underlying mechanism of modified PD mitigating DSS-induced UC, transcriptome sequencing (RNA-seq) analysis was performed to detect differential expression profiles in the colon of NC, DSS and modified PD groups. MA plots and volcano plots of the fold change in gene expression for comparison between each two groups are shown in Fig. 3A, in which we found 2573 differential genes between normal and DSS group, 2019 differential genes between DSS and modified PD group, 1062 differential genes between normal and modified PD group |foldchange| ≥2 and p value ≤0.05 were deemed significantly. Figure 3B displayed a more concrete comparison by showing heatmaps and cluster analysis between each two groups. These preliminary analyses reveal that modified PD indeed elicits therapeutic effects on DSS-induces UC.

To identify the transcriptomic pathways affected by the DSS and modified PD, the gene ontology (GO) analysis and Kyoto encyclopedia of genes and genomes (KEGG) analysis were utilized (Fig. 3C). As shown Fig. 3D, DSS treatment led to activation of Jak/STAT signaling pathway, whereas modified PD reversed this tendency. Additionally, we noticed that level of IL-6 varied with the DSS and modified PD treatment. Thereby these genes and pathways might play important roles in the DSS-induced UC and in therapeutic effect of modified PD.

### Modified PD inhibits DSS-induced activation of IL-6/STAT3 signaling pathway in vitro and in vivo

**Fig. 4:**
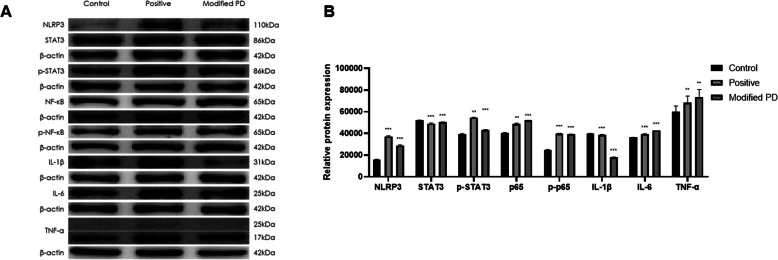
Marker proteins expression of IL-6/STAT3 pathway (A) Western blot analysis of NLRP3, STAT3, p-STAT3, NF-κB, p-NF-κB, p-p65, IL-1β, IL-6 and TNF-α of expression in NCM460 cell. (B) The quantitative analysis for western blot. *: p < 0.05, **: p < 0.01, ***: p < 0.001. Data are expressed as mean ± SEM.

**Fig. 5:**
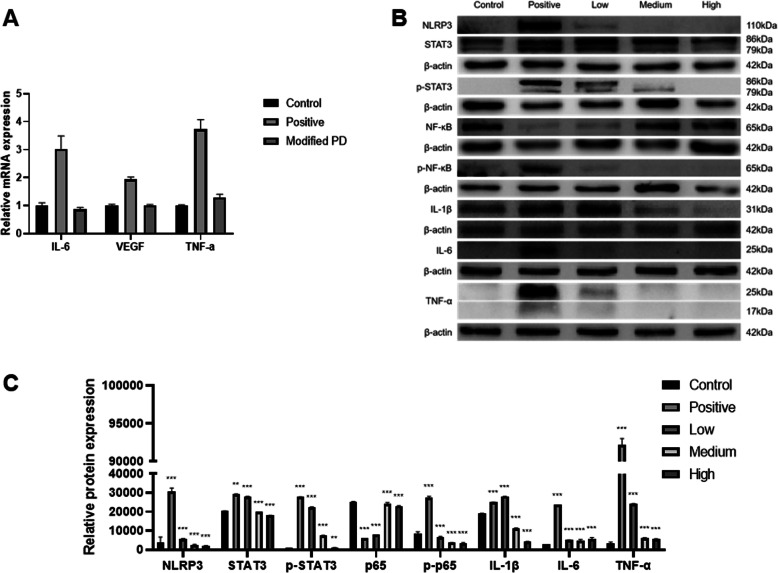
Modified PD mitigated inflammation in DSS-induced UC in vivo. Colon tissues of negative control group, model group (DSS-treated) and modified PD group (DSS-treated with modified PD treatment) were collected. (A) qRT-PCR results indicating modified PD treatment reduced mRNA level of IL-6, VEGF and TNF-α (Modified PD represent high dose modified PD treatment mice herein). (B) Effect of modified PD on the protein expression of NLRP3, STAT3, p-STAT3, NF-κB, p-NF-κB, p-p65, IL-1β, IL-6 and TNF-α. Protein expression levels were analyzed by Western Blot. (C) The quantitative analysis for western blot. *: p < 0.05, **: p < 0.01, ***: p < 0.001. Data are expressed as mean ± SEM.

Previous study has shown that inhibiting or blocking the activation of IL-6/STAT3 signaling pathway can attenuate the colon injury and inflammation in DSS-induced colitis, and RNA-seq analysis also pointed out that the underlying mechanism may related with IL-6/STAT3 pathway. To confirm this, we first selected the NCM460 cell to validate it. As shown in Fig. 4A&B, western blot showed that DSS treatment significantly increased the expression of NLRP3, IL-1β, IL-6 and TNF-α, meanwhile the phosphorylation levels of several marker proteins of IL-6/STAT3 signaling pathway (STAT3 and NF-κB) were also manifested a remarkable upward trend compared to matched controls. However, modified PD could restore this increment. In addition, modified PD treatment significantly inhibited the elevated mRNA expressions of VEGF, IL-6 and TNF-α in colonic tissues of DSS-treated mice, which illustrated that the adverse effects of modified PD might be mediated by the IL-6/STAT3 pathway (Fig. 5A). Moreover, the results of protein levels in colonic tissues were consistent with NCM460 cell (Fig. 5B&C). Taken together, modified PD could downregulate DSS-activated IL-6/STAT3 signaling pathways and suppress inflammatory cytokines production. These findings indicated that IL-6/STAT3 played an important role in DSS-induced acute UC, and modified PD could alleviate oxidative injury in the DSS-induced mouse model of UC.

## Discussion

DSS, a low-molecular weight sulfated polysaccharide, was utilized in inducing epithelial damage and inflammatory response in the colon in experimental mouse model, which could provide signs of acute colitis including weight loss, bloody stools, and diarrhea [15]. Besides, given the massive application of murine colitis model on acute colitis study, we chose C57BL/6 mice to perform the experiments [16–19]. As previously mentioned, the cause and underlying mechanisms of UC remain unclear, but what can be determined is that the chronic relapsing-remitting inflammatory condition recruits proinflammatory cytokines such as interleukin-6 (IL-6), IL-1β, TNF-α and interferon-γ (IFN-γ), thereby resulting in severe colon injury [20]. The conventional treatments for UC including aminosalicylates, corticosteroids, and immune modulators, such as sulfasalazine and glucocorticosteroids, induce remission in only half of patients [21]. However, these chemotherapies can cause serious side effects like vomiting, anemia and generalized edema which can be life-threatening [10]. Due to its obscure etiology, high risk of recurrence, and poor prognosis, UC has become a clinical challenge in terms of treatment. Therefore, studies on the alternative therapies for inflammatory chronic disease, especially UC, have been the sudden explosion of great interest recently.

Traditional Chinese Medicine (TCM) is one of the most developed branches of herbal medicine, which use plants or/and plant extracts for medical treatment. It has been recorded that a number of natural products exhibit effectiveness for the treatment of UC, including Curcumin, Cannabinoids, Andrographis paniculate, Tripterygium wilfordii, et al. [22–24]. Pulsatilla decoction, which consists of namely Radix Pulsatillae, Rhizoma Coptidis, Cortex Phellodendri and Cortex Fraxini, is a TCM formulation derived from a medical book “Shang Han Lun” written by an ancient Chinese physicians Zhang Zhongjing about 1800 years ago. Several trials have shown that the prescription exerted prominent anti-inflammatory effect, especially on enteritis and bacillary dysentery [25]. Based on the classical prescription and our clinical experience, we improved the proportion of ingredients in Pulsatilla decoction, and added another herbal plant, roasted Rhizoma Atractylodis Macrocephalae.

**Fig. 6:**
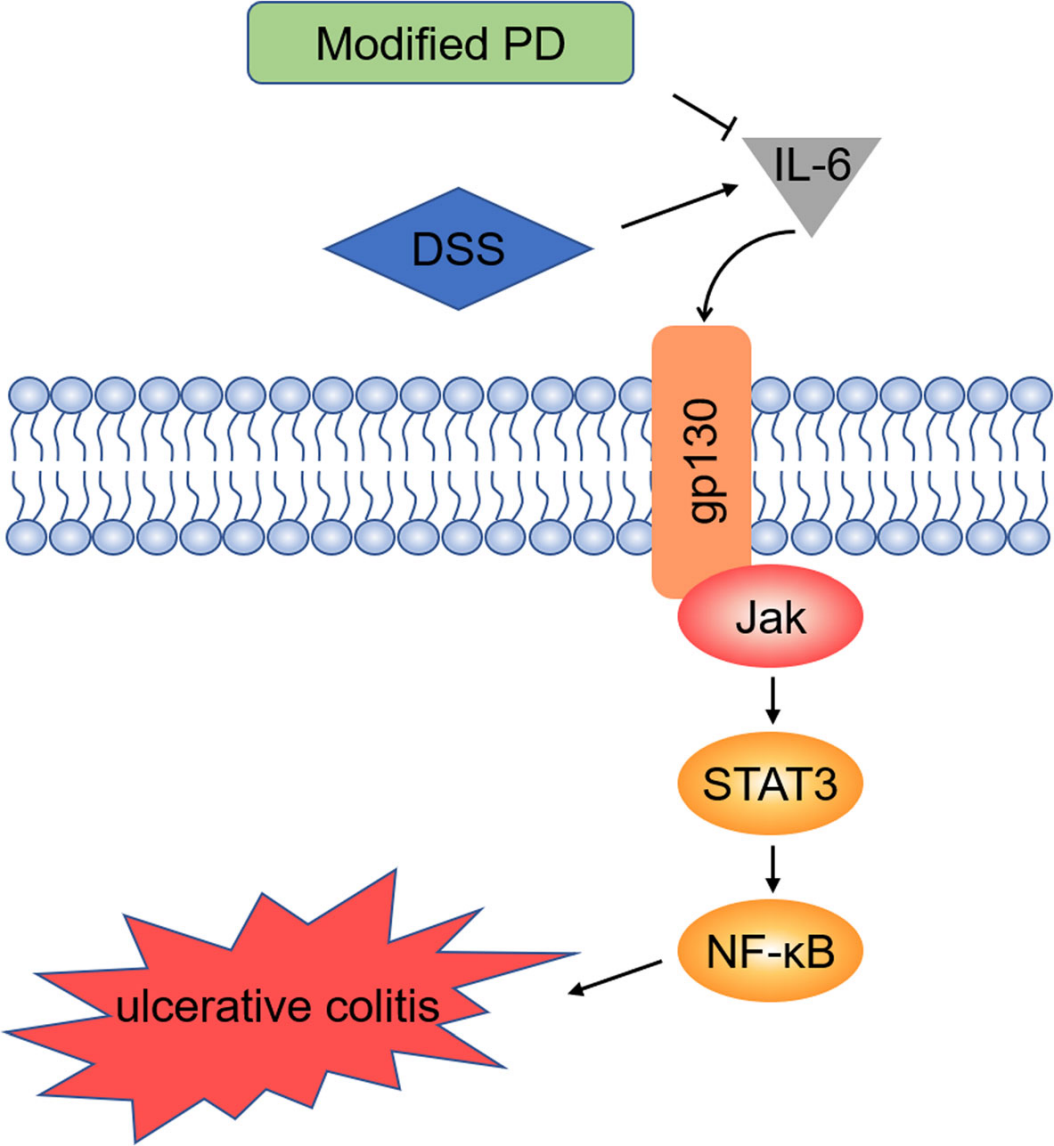
Modified PD inhibits DSS-induced ulcerative colitis in mice through IL-6/STAT3 pathway

In our study, we examined whether modified PD alleviated the severity in tissue affected by colitis, the results showed that treatment with modified PD improved the extent of damage in colon suffering UC. Besides, transcriptome sequencing indicated that IL-6/STAT3 signaling pathway may be involved in the anti-inflammatory mechanism of modified PD. Western blot verified the decreased expression of IL-6 and p-STAT3 in colon tissue. Meanwhile, the expression of NF-κB, NLRP3, IL-1β and TNF-α were diminished. Through IL-6/STAT3 signaling pathway, modified PD could inhibit the increased inflammatory response and reduce the severity of colitis lesions (Fig. 6). The multi-target effect of TCM formulation lead us to wonder whether modified PD functions by influencing other signaling pathway. KEGG pathway classification from transcriptome sequencing reveal that TLR4/MyD88 signaling is one of the most significantly enriched pathways, which play a vital role mediating inflammation response. TLR4 mainly recognizes pathogen-associated molecules and after being stimulated, TLR4 recruits and activates downstream IRAK, ARAK2 and TRAF6, eventually regulating MAPK, IRF5, NF-κB and as a result, terminal inflammatory factors IL-1β and TNF-α are released [26]. Several researches have showed that various TCM formulations and compounds could alleviate inflammatory bowel diseases through TLR4/MyD88 pathway [27–29], nonetheless whether modified PD takes effect through via this signaling still needs further investigation.

Our work indicated the anti-colitis potential of modified PD in vitro and in vivo, shedding light on UC interruption through utilizing modified PD. Further animal experiments show that modified PD can significantly improve the symptoms of stool with pus and blood and weight loss in the acute stage of inflammation, and significantly reduce the occurrence of IBD-CRC when used to treat AOM/DSS-induced IBD-CRC. The mechanism remains to be further elucidated.

## Conclusions

In summary, we demonstrated that modified PD could inhibit the increased inflammatory response and reduce the severity of colitis lesions through IL-6/STAT3 signaling pathway. Meanwhile, modified PD decreased the expression of NF-κB, NLRP3, IL-1β and TNF-α. These results suggest that the anti-colitis potential of modified PD in vitro and in vivo, shedding light on UC interruption through utilizing modified PD.

## Methods

### Cell culture

NCM460 cells were purchased from Xunian Biological Technology Co., Ltd. (Shanghai, China). Cells were maintained in Dulbecco’s Modified Eagle Medium (Gibco, Grand Island, NY) with 10% fetal bovine serum (FBS) (Gibco, Grand Island, NY), and penicillin and streptomycin (P/S) solution (Sigma, St. Louis, MO) in a 37 °C incubator with humidified atmosphere of 5% CO_2_.

### Reagents

Chinese herbal medicine was purchased from Nanjing Hospital of TCM affiliated to Nanjing University of Chinese Medicine, decoctioned to the appropriate concentration in the laboratory, stored at 4 °C after dispensing, then used for gavage. Common names and Latin names of ingredients in modified PD are presented in Table 1. TNF-a (#11948), IL-1β (#12703), IL-6 (#12153), STAT3 (#4904), phospho-STAT3 (#9145), NF-κB (#8242), phospho-NF-κB (Ser468) (#3039), NLRP3 (#15101) and β-actin (#4970) were purchased from Cell Signaling Technology (Danvers, MA). Dextran sulphate sodium (DSS; 36–50 kDa) was obtained from MP Biomedicals (Illkirch, France).

### Cell viability assay

The cells were seeded into 96-well plates and treated with drug at a serious of concentrations for 24 h and 48 h. After treatments, cells were incubated with MTT (Solarbio, Shanghai, China) for 4 h at 37 °C. Then the supernatant was removed, and the formazan crystals were dissolved in 200 μL dimethyl sulfoxide. Finally, the optical density was measured at 570 nm with a microplate plate reader (Thermo Fisher Scientific, Inc., USA).

### Mouse model

Eight-weeks-old female C57BL/6 mice were purchased from Nanjing Qinglongshan Animal Breeding Base (Nanjing, China). All animals were reared in an SPF-level laboratory (temperature 24–25 °C, humidity 70–75%, with a 12 h light/dark lighting regimen) and were fed a standard diet of pellets and water ad libitum. Animal welfare and experimental procedures were carried out strictly in accordance with the Guide for the Care and Use of Laboratory Animals (National Institutes of Health, the United States) and the IACUC protocols of our university (approval no. ACU170501). Mice were randomly assigned to 5 groups, 9 mice per group and 45 mice in total: negative control group (mice received drinking water and saline), model group (mice received DSS in drinking water only), low dose modified PD group (3.185 g/kg together with DSS), medium dose modified PD group (6.37 mg/kg together with DSS) and high dose modified PD group (12.74 mg/kg together with DSS).

Colitis was induced by providing 2.5% DSS (w/v) to mice in drinking water for 7 days, and were simultaneously given modified PD of different doses since the first day [10]. Body weight was measured daily and the animals were sacrificed at day 11 by cervical dislocation, and their colons were collected. The colon was fixed in 10% formalin for at least 24 h for further histopathological assessment.

### RNA extraction and quantitative real-time PCR (qRT-PCR)

Total RNA was extracted from colon tissues by using Trizol regent (Invitrogen, US) according to the manufacturer’s procedural guidelines, and cDNA was synthesized by using HiScript RT SuperMix for qPCR kit (Vazyme, China). Then quantitative real-time PCR was performed by using iTaq SYBR Green Supermix With ROX kit (Bio-Rad, US). The specific primers used for detecting genes are listed in Table 2. All quantitative real-time PCR experiments were performed with LightCycler 96 System (Roche, Germany). Relative expression of target genes was normalized to GPADH, analyzed by 2^-ΔΔCt^ method and given as ratio compared with the control. The primer sequences are shown as follow.

### High-throughput transcriptomic sequencing

RNA samples were sent to the Weifen Biotech (Anhui, China) for RNA-seq. Briefly, total RNAs were isolated from triplicates of colon tissues at control group, DSS group and modified PD group (here we selected the high dose modified PD group). Three biological repeats were included for each group. mRNA was extracted from the total RNA after removing 16 and 23 s rRNAs and then were pooled together for cDNA synthesis and sequencing. After generating the clusters, library sequencing was performed on an Illumina HiSeq2000 platform, to create paired-end reads with a length of 150 bp. Gene ontology and KEGG pathway analysis were performed using DAVID.

### Western blot

Whole cellular or tissue proteins were extracted with Pierce RIPA Buffer (Thermo Scientific, US) and protease inhibitor cocktail (Yeasen, Shanghai, China), then lysates were transferred to a 1.5-ml microcentrifuge tube and centrifuged at 12000 rpm for 20 min at 4 °C. The supernatant was retained and the protein concentration was normalized to equal level by using BCA Protein Assay kit (Thermo Fisher, USA). The extracts were separated by SDS-PAGE and then transferred to 0.45 μm immobilon-P transfer membrane (Millipore, Bedford, MA). Membranes were blocked with 5% skim milk for 1 h followed by incubation with a primary antibody at 4 °C overnight. Then they were washed and treated with an HRP labeled secondary antibody at 37 °C for 2 h. Immunoblots were visualized with the High-sig ECL Western Blot Substrate (Tannon, Shanghai, China).

### Histological analysis

Mice colon tissue was fixed with a sufficient amount of 10% formalin for 24 h. Tissue sections were prepared by material extraction, dehydration, wax dipping, embedding, sectioning, HE staining, and coverslipping. The sections were scanned by digital scanning system and electronic sections were stored and analyzed on the computer.

The specific scoring method of histological damage is as follows: the pathological morphological feature in the visual field is normal intestinal mucosa, 0 point is scored; the pathological morphological feature is mild inflammation and edema of the mucosal layer, and the 1/3 crypt in the basal part disappears, 1 point is scored; It is moderate inflammation of the mucosal layer, 2/3 of the crypts at the base part disappear, 2 points are scored; pathological features are moderate inflammation of the mucosa layer, the crypts completely disappear, but the epithelial layer is still intact, 3 points are scored; pathological features are mucosa inflammation, which was severe in the stroma, submucosa, and myometrium, and the crypts and epithelium disappeared, 4 points were scored [30–32].

### Statistical analysis

All data were presented as means ± standard deviation (SEM). One-way analysis of variance (ANOVA) was used to evaluate the data between two experimental groups. For all analyses, p < 0.05 were considered statistically significant. They were undertaken using the GraphPad Prism software (GraphPad Software Inc., Avenida, CA).

## Data Availability

The datasets used and/or analyzed in the current study are available from the corresponding author on reasonable request.

## Abbreviations

IBD: Inflammatory bowel disease
UC: Ulcerative colitis
CD: Crohn’s disease
PD: Pulsatillae decoction
DSS: Dextran sodium sulfate
CRC: Colorectal cancer
TNF: Tumor necrosis factor
IFX: Infliximab
ADA: Adalimumab
qRT-PCR: Quantitative real-time PCR
GO: The gene ontology
KEGG: Kyoto encyclopedia of genes and genomes
